# Cytotoxicity, antimicrobial and antioxidant activity of eight compounds isolated from *Entada abyssinica* (Fabaceae)

**DOI:** 10.1186/s13104-017-2441-z

**Published:** 2017-03-06

**Authors:** Jean P. Dzoyem, Raduis Melong, Armelle T. Tsamo, Alembert T. Tchinda, Deccaux G. W. F. Kapche, Bonaventure T. Ngadjui, Lyndy J. McGaw, Jacobus N. Eloff

**Affiliations:** 1 Phytomedicine Programme, Department of Paraclinical Sciences, Faculty of Veterinary Science, University of Pretoria, Private Bag X04, Onderstepoort, Pretoria, 0110 South Africa; 2 Department of Biochemistry, Faculty of Science, University of Dschang, P.O. Box 67, Dschang, Cameroon; 3 Department of Organic Chemistry, Faculty of Science, University of Yaoundé I, P.O. Box 812, Yaoundé, Cameroon; 4 Department of Chemistry, Higher Teachers’ Training College, University of Yaoundé I, P.O. Box 47, Yaoundé, Cameroon; 5 Institute of Medical Research and Medicinal Plants Studies (IMPM), Center of Medicinal Plants Studies and Traditional Medicine (CRPMT), P.O. Box 6163, Yaoundé, Cameroon

**Keywords:** Cytotoxicity, Antibacterial, Free radical scavenging, *Entada abyssinica*

## Abstract

**Background:**

*Entada abyssinica* is a plant traditionally used against gastrointestinal bacterial infections. Eight compounds including three flavonoids, three terpenoids, a monoglyceride and a phenolic compound isolated from *E. abyssinica* were investigated for their cytotoxicity, antibacterial and antioxidant activity.

**Results:**

Compounds **7** and **2** had remarkable activity against *Salmonella typhimurium* with the lowest respective minimum inhibitory concentration (MIC) values of 1.56 and 3.12 µg/mL. The antioxidant assay gave IC_50_ values varied from 0.48 to 2.87 μg/mL in the 2,2-diphenyl-1-picrylhydrazyl (DPPH) assay, from 2.53 to 17.04 μg/mL in the 2,2′-Azino-bis (3-ethylbenzothiazoline-6-sulphonic acid) diammonium salt (ABTS) assay and from 1.43 to 103.98 µg/mL in the FRAP assay. Compounds had relatively low cytotoxicity (LC_50_ values ranging from 22.42 to 80.55 µg/mL) towards Vero cells. Ursolic acid had the most potent cytotoxicity against THP-1 and RAW 264.7 cells with LC_50_ values of 9.62 and 4.56 μg/mL respectively, and selectivity index values of 7.32 and 15.44 respectively.

**Conclusion:**

Our findings suggest that among the terpenoid and flavonoid compounds studied, entadanin (compound **7**) possess tremendous antibacterial activity against *S. typhimurium* and could be developed for the treatment of bacterial diseases.

## Background

Oxidative stress occurs when there is excessive free radical production and/or low antioxidant defense, which leads to many pathophysiological conditions in the body [1]. To neutralize free radicals and protect the body against oxidative damage, different antioxidants which are present in normal physiological conditions are able to counteract the production of reactive oxygen species. Free radicals are known to be the main cause of various diseases such as cancer and bacterial diseases. The development of resistance to multiple drugs in microbes and tumor cells has become a major public health threat [2, 3]. Cancer is one of the leading causes of death in most well developed countries. A large body of evidence has determined that relationships exist among certain bacteria and cancers [4]. Because of the resistance that pathogenic microorganisms and malignant cells build against current antibiotics and anticancer drugs, there is great interest in the search for new therapeutic agents. Thus, in recent years there has been increased use of plants and their derivatives as an alternative modality in the treatment of various diseases, including cancer and infections caused by microorganisms [5]. Unlike synthetic drugs, bioactive natural products can have a beneficial effect on the whole organism and with less toxic effects. Therefore, natural products will continue to be extremely important as sources of discovery of new medicinal agents. *Entada abyssinica* A.Rich (Fabaceae) is a tree widely spread in tropical Africa. It is traditionally used to treat coughs, rheumatism, bronchitis, abdominal pains, diarrhoea and fever and to prevent miscarriage [6, 7]. Some pharmacological properties of *E. abyssinica* have been previously reported, including anti-inflammatory, antimicrobial and antioxidant [8–10]. Previous phytochemical screening of *E. abyssinica* indicated the presence of flavonoids, terpenoids and kolavic acid derivatives [11–13]. Considering the vast potential of plants as sources of antimicrobial and anticancer drugs, the objective of this study was to examine the possible antiproliferative, antimicrobial and antioxidant activity of terpenoid and flavonoid compounds isolated from *E. abyssinica*.

## Methods

### Chemicals and compounds

Gentamicin was obtained from Virbac, South Africa. Sodium carbonate was provided by Holpro Analytic, South Africa. Dulbecco’s Modified Eagle Medium (DMEM) and Fetal calf serum (FCS) were purchased from Highveld Biological, South Africa. Whitehead Scientific, South Africa provided trypsin and Phosphate buffered saline (PBS). *p*-iodonitrotetrazolium violet (INT), doxorubicin, 2,2-diphenyl-1-picrylhydrazyl (DPPH), 3-(4,5-dimethylthiazol-2-yl)-2,5-diphenyltetrazolium bromide (MTT), puromycin, 2,2′-Azino-bis (3-ethylbenzothiazoline-6-sulphonic acid) diammonium salt (ABTS), dimethyl sulfoxide (DMSO), were provided by Sigma-Aldrich St. Louis, MO, USA, while Müller-Hinton agar and broth were from Sigma-Aldrich, India.

**Fig. 1:**
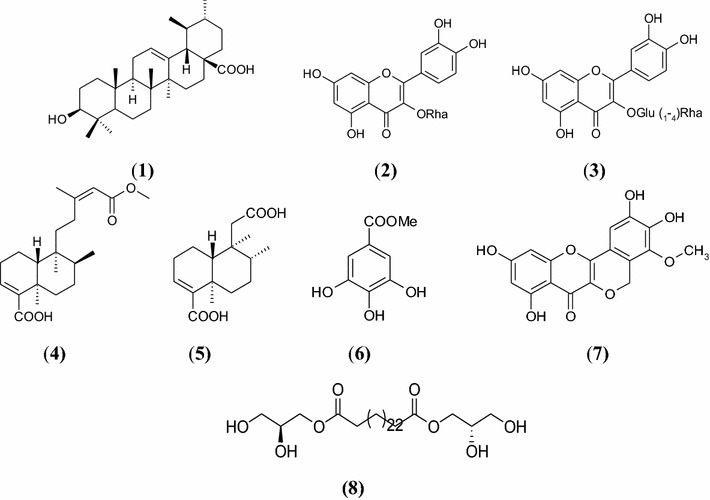
Chemical structures of ursolic acid (**1**), quercetin-3-*O*-α-l-rhamnoside or quercitrin (**2**), quercetin 3-*O*-β-d-glucosyl (1→4)-α-l-rhamnoside (**3**), (8*S*)-kolavic acid 15-methyl ester (**4**), 13,14,15,16-tetranor-3-clerodene-12,18-dioic acid (**5**), methyl gallate (**6**), entadanin (**7**), bis-[(*S*)-(2,3-dihydroxypropyl)] hexacosanedioate (**8**)

Naturally occurring compounds studied in this work were isolated from the leaves and stembark of *Entada abyssinica.* The leaves of *E. abyssinica* was collected in May 2012 at Balatchi (Mbouda), in the West region of Cameroon, and identified by Mr. Victor Nana (plant taxonomist) of the National Herbarium of Cameroon, Yaoundé, where a voucher specimen is deposited under reference number 32436/HNC. Compounds studied included: ursolic acid (**1**), quercetin-3-*O*-α-l-rhamnoside or quercitrin (**2**), quercetin-3-*O*-β-D-glucosyl (1→4)-α-l-rhamnoside (**3**), (8*S*)-kolavic acid 15-methyl ester (**4**), 13,14,15,16-tetranor-3-clerodene-12,18-dioic acid (**5**), methyl gallate (**6**), entadanin (**7**), bis-[(*S*)-(2,3-dihydroxypropyl)] hexacosanedioate (**8)**. We previously described their isolation procedure and their structure elucidation [14]. Chemical structures are shown in Fig. 1.

### Antimicrobial activity

The six bacterial strains included: *Pseudomonas aeruginosa* ATCC 27853, *Bacillus cereus* ATCC 14579, *Staphylococcus aureus* ATCC 29213, *Escherichia coli* ATCC 25922, *Salmonella typhimurium* ATCC 14028 and *Enterococcus faecalis* ATCC 29212. The antimicrobial activity was evaluated by determining the minimal inhibitory concentration (MIC) by the rapid *p*-iodonitrotetrazolium violet (INT) microdilution method as previously described [15].

### Antioxidant assays

#### ABTS radical assay

The antioxidant activity by ABTS was assessed according to the method previously described [16].

#### DPPH assay

The DPPH radical-scavenging activity was assessed by the method previously described [16].

#### Ferric reducing antioxidant power (FRAP) assay

The antioxidant activity by the ferric reducing antioxidant power (FRAP) was assessed according to the method previously described with slight modifications [16].

### Cytotoxicity assay

#### Cell culture

Cancer cell lines including human monocytic THP-1 and murine macrophage RAW 264.7 cells and the normal mammalian Vero monkey kidney cell line were obtained from the American Type Culture Collection (Rockville, MD, USA). They were maintained in DMEM under standard cell culture conditions at 37 °C and 5% CO_2_ in a humidified environment.

#### MTT assay

The 3-(4,5-dimethylthiazol-2-yl)-2,5-diphenyltetrazolium bromide (MTT) assay was used to determine the cytotoxicity of the compounds as previously described [15]. The selectivity index (SI) values to identify selective anti-cancer cell activity were calculated by dividing the LC_50_ values of normal Vero cells by the LC_50_ of cancer cells.

### Statistical analysis

Experiments were performed three times and values were expressed as mean ± standard deviation. Differences between IC_50_ values were analysed for statistical significance using ANOVA and compared using the Fisher’s least significant difference (LSD) at 5% interval confidence.

## Results

The structures of compounds isolated from *E. abyssinica* (Fig. 1) were established based on spectroscopic data and direct comparison with previously published data. Their antibacterial activity results are presented in Table 1. The overall results showed that compounds presented variable antibacterial activity with MIC values ranged between 1.56 and 100 µg/mL. Gram-positive bacteria were more sensitive than the Gram-negative bacteria. In particular *S. typhimurium* had the highest susceptibility to the compounds with the lowest MIC values of 1.56 µg/mL followed by *B. cereus* (MIC values of 6.25 µg/mL). Compounds **7** and **2** had the most potent antibacterial activity against *S. typhimurium* with MIC values of 1.56 and 3.12 µg/mL respectively and moderate activity against *S. aureus* (MIC = 12.5 µg/mL). Similarly, compound **1** (ursolic acid) had significant activity against *B. cereus* (MIC = 6.25 µg/mL).

**Table 1:** Antibacterial activity of eight compounds isolated from *Entada abyssinica* (MIC in µg/mL)

Compounds	MIC (µg/mL)					
	*Sa*	*Bc*	*St*	*Pa*	*Ef*	*Ec*
**1**	12.5	6.25	100	–	–	–
**2**		12.5	3.12	50	25	50
**3**	25	50	25	50	50	25
**4**	25	25	100	–	–	–
**5**	–	–	–	–	–	–
**6**	50	50	25	–	–	–
**7**	12.5	25	1.56	25	–	12.5
**8**	–	–	–	–	–	–
Gentamicin	0.5	0.5	2	0.25	0.25	1

– 100 µg/mL. *Sa Staphylococcus aureus, Ef Enterococcus faecalis, Bc Bacillus cereus, Ec Escherichia coli, Pa Pseudomonas aeruginosa, St Salmonella typhimurium*

For the antioxidant activity, samples were tested at several concentrations, then from the dose–response activities, the IC_50_ values were obtained and are presented in Table 2. The IC_50_ values for the different compounds ranged from 0.48 to 2.87 μg/mL in the DPPH assay, from 2.53 to 17.04 μg/mL in the ABTS assay and from 1.43 to 103.98 µg/mL in the FRAP assay.

**Table 2:** Antioxidant activity of eight compounds isolated from *Entada abyssinica*

Compounds	DPPH (IC_50_, µg/mL)	ABTS (IC_50_, µg/mL)	FRAP (µmol FeSO_4_/g)
**1**	2.87 ± 1.19^a^	7.04 ± 1.29^a^	1.43 ± 0.80^a^
**2**	0.9 ± 0.06^b^	3.53 ± 0.39^b^	76.01 ± 1.10^b^
**3**	2.08 ± 0.19^a^	17.04 ± 0.26^c^	75.34 ± 1.06^b^
**4**	–	–	1.93 ± 0.14^a^
**5**	–	–	5.09 ± 0.40^c^
**6**	0.48 ± 0.02^c^	2.53 ± 0.49^d^	103.98 ± 13.70^d^
**7**	1.12 ± 0.10^c,d^	4.13 ± 0.10^e^	72.41 ± 2.02^b,e^
**8**	–	–	22.98 ± 4.29^f^
Trolox	8.71 ± 2.03^e^	10.38 ± 2.4^a,f^	nd
Ascorbic acid	3.44 ± 1.9^a,f^	4.15 ± 1.21^d,e^	nd

Data represent the mean ± SD of three independent experiments; values with different letters are significantly different at p < 0.05

*nd* not determined, – 100 µg/mL

For the cytotoxicity, the LC_50_ values were determined and the selectivity index (SI) values were calculated and presented in Table 3. A perusal of Table 3 shows that compounds were less toxic than the positive control (LC_50_ values ranging from 22.42 to 80.55 µg/mL) towards the Vero cells suggesting relative lack of cytotoxicity. The anti-proliferative activity against cancer cell lines showed that compounds had LC_50_ values ranging from 9.62 to >100 µg/mL and the SI ranged from 0.84 to 7.32 on THP-1 cells. For RAW 264.7 cells, the LC_50_ values varied from 4.56 to 86.55 µg/mL and the SI ranged from 0.81 to 15.44. Compound **1** had the most potent cytotoxicity against THP-1 and RAW 264.7 cells with LC_50_ values of 9.62 and 4.56 μg/mL respectively.

**Table 3:** Cytotoxicity (LC_50_ in µg/mL) of eight compounds isolated from *Entada abyssinica* and their selectivity index (SI) values against normal and cancer cell lines

Compounds	Vero LC_50_	THP-1		RAW 264.7	
		LC_50_	SI	LC_50_	SI
**1**	22.42 ± 2.48^a^	9.62 ± 0.59^a^	7.32	4.56 ± 0.020^a^	15.44
**2**	44.83 ± 2.83^b^	–	nd	16.44 ± 0.20^b^	4.28
**3**	53.76 ± 2.05^c^	–	nd	41.90 ± 0.43^c^	1.68
**4**	47.46 ± 0.63^b,d^	49.78 ± 3.03^b^	1.41	52.30 ± 1.30^d^	1.35
**5**	41.91 ± 1.85^b,e^	21.81 ± 1.11^c^	3.23	16.10 ± 1.00^b^	4.37
**6**	30.58 ± 3.09^f^	75.00 ± 1.68^d^	0.94	36.92 ± 1.27^e^	1.91
**7**	55.65 ± 0.30^c^	84.28 ± 3.30^e^	0.84	19.12 ± 0.25^f^	3.68
**8**	80.50 ± 4.83^g^	65.00 ± 6.88^d,f^	1.08	86.55 ± 4.61^g^	0.81
Doxorubicin	9.35 ± 0.66^h^	–	nd	0.5 ± 0.00^h^	nd
Puromycin	5.32 ± 0.90^i^	0.4 ± 0.02^g^	176.03	1.15 ± 0.17^i^	61.23

Data represent the mean ± SD of three independent experiments; values with different letters are significantly different at p < 0.05

*nd* not determined, *–* 100 µg/mL

## Discussion

The antibacterial potential ranged from significant to weak activity. Ursolic acid is an ubiquitous compound that can be isolated from many medicinal plants and its antibacterial activities are well documented. It has been reported to be active against many bacterial species, particularly Gram-positive species, inhibiting bacterial growth of *S. aureus* with a MIC value of 4 µg/mL [17, 18]. It is noteworthy that the activity of compound **7** (entadanin) against *S. typhimurium* was comparable to the standard gentamicin. Quercitrin is a quercetin-related flavonoid and previous studies have shown that quercetin and its glycosides quercetin-3-O-α-l-arabinopyranoside and quercetin-3-O-β-d-arabinopyranoside have strong antibacterial activity against the Gram-positive *S. aureus*, and the Gram-negative *P. aeruginosa* and *E. coli* with MIC values ranged from 0.093 to 0.37 µg/mL [19].

The antioxidant activity of compounds can be determined in vitro by hydrogen atom transfer (HAT) method and single electron transfer (SET) method. HAT methods measure the capacity of an antioxidant to scavenge free radicals by hydrogen donation to form a stable compound. SET methods determine the ability of the antioxidant to transfer one electron to reduce compounds including metals, carbonyls and radicals [20]. The FRAP assay involves the SET method, while the DPPH and ABTS assays involve both methods, but predominantly the SET method [21]. In this study, the antioxidant activity of compounds was determined using the free radical 2,2′-azino-bis (3-ethylbenzothiazoline-6-sulphonic acid) diammonium salt (ABTS), 2,2-diphenyl-1-picrylhydrazyl (DPPH) and the ferric reducing antioxidant power (FRAP) assays. The use of at least two different assays in evaluating antioxidant activity of plant products has been recommended by Moon and Shibamoto [22].

The antioxidant activity revealed that, the IC_50_ values of compounds **7**, **6** and **2** were significantly different from the IC_50_ values of ascorbic acid and trolox, which are standard antioxidant agents used as positive controls. The capacity of flavonoids to act as antioxidants in vitro has been previously studied [23]. However, the antioxidant activity of entadanin, a new peltogynoid is here reported for the first time.

In order to ascertain the likely safety of compounds for their potential use, a standard cell-based toxicity assay was performed for cytotoxicity evaluation against Vero monkey kidney cells. In addition, the anti-proliferative activity was assessed on two cancerous cell lines (THP-1 and RAW 264.7). According to the in vitro cytotoxic activity criteria suggested by Syarifah et al. [24], a compound is considered as weakly active if the LC_50_ ≥ 50 µg/mL, moderately active for 10 µg/mL < LC_50_ < 50 µg/mL and significantly active if LC_50_ ≤ 10 µg/mL). Considering this cut-off, the activity obtained with compound **1** (ursolic acid) against THP-1 and RAW 264.7 cells could be considered significant. Ursolic acid is a natural pentacyclic triterpenoid carboxylic acid present in a wide variety of plants, including apples, basil, bilberries, cranberries, peppermint, rosemary and oregano [25]. Several pharmacological effects of ursolic acid including anti-proliferative properties have been reported in a number of experimental systems [26]. It should be noted that this is the first report on the biological activity of compound **7**, a cyclic homoflavonoid (entadanin), and compound **8** (bis-[(*S*)-(2,3-dihydroxypropyl)] hexacosanedioate).

## Conclusion

Our findings suggest that among the terpenoid and flavonoid compounds studied, entadanin (compound **7**), whose activities are reported here for the first time, possesses extremely interesting antibacterial activity against *S. typhimurium.* Therefore, this compound could be investigated further for its potential use in the treatment of bacterial diseases, especially gastrointestinal infections caused by *S. typhimurium.*

## Abbreviations

FCS: fetal calf serum
PBS: phosphate buffered saline
ABTS: 2,2′-azino-bis (3-ethylbenzothiazoline-6-sulphonic acid) diammonium salt
DPPH: 2,2-diphenyl-1-picrylhydrazyl
MTT: 3-(4,5-dimethylthiazol-2-yl)-2,5-diphenyltetrazolium bromide
DMSO: dimethyl sulfoxide
INT: p-iodonitrotetrazolium violet
MHB: Muller Hinton broth
DMEM: Dulbecco’s Modified Eagle Medium
FRAP: ferric reducing antioxidant power
TPTZ: tripyridyl triazine
